# Traditional Chinese medicine for mild cognitive impairment: A protocol for systematic review and network meta-analysis

**DOI:** 10.1097/MD.0000000000022187

**Published:** 2020-09-11

**Authors:** Haiyan Wang, Haiyang Yu, Kai Song, Fanjie Xiong, Hong Zhang

**Affiliations:** a College of Acupuncture and Tuina, Chengdu University of Traditional Chinese medicine, Chengdu, Sichuan Province, China; b Department of Acupuncture and Moxibustion, Affiliated Hospital of Gansu University of Traditional Chinese Medicine, Lanzhou, Gansu Province, China; c Department of Traumatic Orthopedics, Affiliated Hospital of Gansu University of Traditional Chinese medicine, Lanzhou, Gansu Province, China.

**Keywords:** mild cognitive impairment, protocol, systematic review, traditional Chinese medicine therapies

## Abstract

**Background:**

Mild cognitive impairment (MCI) is an intermediate stage between normal aging and Alzheimer disease, which is the most common form of dementia in the world. In clinical practice, traditional Chinese medicine (TCM) interventions have been administered for MCI, However, there is still uncertain about what strategy of TCM interventions treatment should be preferred in clinical practice. This study aims to evaluate the efficacy and acceptability of different TCM therapies through systematic review and network meta-analysis.

**Methods:**

According to the strategy, the authors will retrieve a total of 7 electronic databases by August 2020, including PubMed, the Cochrane Library, EMbase, China National Knowledge Infrastructure, China Biological Medicine, Chongqing VIP, and Wan-fang databases. After a series of screening, 2 researchers will use Aggregate Data Drug Information System and Stata software to analyze the data extracted from the randomized controlled trials of TCM therapies for MCI. The primary outcome of this study is the improvement of cognitive function and the secondary outcome is the activities of daily living, clinical efficacy, and adverse events, and the quality of the evidence will be evaluated using the Grading of Recommendations Assessment, Development and Evaluation instrument.

**Results:**

This study will provide a reliable evidence for the selection of TCM therapies in the treatment of MCI.

**Conclusion:**

This study will generate evidence for different TCM therapies for MCI and provide a decision-making reference for clinical research.

**Ethics and dissemination:**

This study does not require ethical approval. The results will be disseminated through a peer-reviewed publication.

**OSF registration number:**

DOI 10.17605/OSF.IO/JV9KG.

## 1 Introduction

Mild cognitive impairment (MCI) is an intermediate stage between normal aging and dementia. In MCI, there is an objective cognitive decline, but independence in daily activities is preserved.^[1]^ MCI represents a significant risk factor for the development of dementia and is the primary target for early detection and management of dementia.^[2–4]^ Population-based studies have found that the prevalence of MCI in elderly individuals (≥65 years) is 10% to 20%, with 5% to 10% of patients progressing to Alzheimer disease (AD) each year.^[5–7]^ It is estimated that the prevalence of MCI is significantly increasing worldwide, and MCI increases the risk of progression to dementia.^[8]^

Currently, there is no approved pharmacological treatments for MCI.^[9]^ The results of systematic reviews and meta-analyses evaluating the efficacy of acetylcholinesterase inhibitors (AChEIs), including donepezil, rivastigmine, and galantamine, show that there is no convincing evidence that AChEIs have an effect on cognitive test scores or the progression of MCI.^[10,11]^ Moreover, in the AChEI groups, there were in-creased risks of adverse events, including diarrhea, nausea, vomiting, muscle spasms or leg cramps, insomnia, headache, and abnormal dreams. There is some evidence suggesting that traditional Chinese medicine (TCM) interventions such as acupuncture, moxibustion, Chinese herbal medicines, physical exercise, and tai chi (TC) might be beneficial for patients with MCI. However, there is currently no established TCM treatment method for MCI.

Acupuncture treatment is widely used in Asia and can be considered as an alternative treatment for MCI.^[12]^ In recent years, a number of basic and clinical studies have provided evidence that acupuncture is beneficial for the treatment of dementia or MCI, and the literature suggests that acupuncture might improve cognitive function and changes in the activities of daily living (ADLs) in patients with cognitive impairment and dementia.^[13–15]^ Animal studies have reported that acupuncture may have effects on multiinfarct dementia and AD via improving memory ability.^[16,17]^

Moxibustion is a form of TCM that has been widely used in East Asia for thousands of years.^[18]^ Moxibustion imparts both heat stimulation via infrared radiation and the pharmacological actions of its herbal components.^[19,20]^ It regulates a multidimensional network that includes the nervous, endocrine, and immune systems, all of which play important roles in maintaining homeostasis, potentially exerting significant therapeutic effects.^[21]^ Various clinical trials and animal studies have been conducted to investigate the benefits and mechanisms of moxibustion for preventing and treating MCI.^[22,23]^

Chinese herbal medicine (CHM) has a long history for treating memory disorders too. Many CHMs, such as Herba cistanches and Polygonum multiflorum, have shown positive therapeutic effects on cognitive impairment.^[24,25]^ CHMs are usually used in combination; a placebo-controlled randomized trial showed that the CHM could improve or maintain the general cognitive function of patients with amnestic mild cognitive impairment (AMCI) through a 2-year treatment.^[26]^

TC combined with both Chinese martial art and health regimens into a common set of core principles, movements, and exercises.^[27]^ Over the last 10 years, TC has gained popularity as a therapeutic physical activity for elders with MCI.^[28]^ A number of nonrandomized and randomized clinical trials have shown the therapeutic effects of TC on cognition. Some reviews have explored the generalized correlation between TC and cognitive function.^[29,30]^

In spite of its long history of use and clinical and experimental support, the effects of TCM for MCI have not been fully validated. In addition, systematic reviews and meta-analyses have not been done yet. Thus, this systematic review and meta-analysis aims to assess the effects and safety of TCM therapies on symptoms of MCI.

## 2 Methods

### 2.1 Protocol and registration

This protocol follows the Preferred Reporting Items for Systematic Reviews and Meta-Analyses Protocols (PRISMA-P) guidelines.^[31]^ The NMA protocol has been registered on Open Science Framework (OSF) platform (https://osf.io/jv9hg/), registration number:DOI 10.17605/OSF.IO/JV9KG.

### 2.2 Eligibility criteria

The participant (P), intervention (I), comparator (C), outcome (O), and study design (S) are the 5 main factors determining the inclusion and exclusion criteria of this research.

#### 2.2.1 Type of participant

Participants with all types of MCI, including AMCI, non-aMCI, and vascular MCI, who meet the diagnostic criteria of MCI will be included. These criteria include the Diagnostic and Statistical Manual of Mental Disorders, International Classification of Diseases, Diagnostic criteria from the International Working Group on Mild Cognitive Impairment, Diagnostic criteria from the MCI Working Group of the European Consortium on Alzheimer's Disease, and the Chinese Classification of Mental Disorders. There is no restriction on age, gender, ethnicity, or economic status of the enrolled participants.

#### 2.2.2 Type of interventions and comparators

Interventions in the treatment group will include any kinds of TCM treatment that include acupuncture, moxibustion, CHM, and TC, etc. We also include TCM in combination with other conservative treatments, such as cognitive rehabilitation and oral drugs. However, combined interventions consisting of 3 or more therapies or with potential safety problems will be excluded. Control interventions will include no treatment, waiting list, oral drugs, cognitive rehabilitation, sham acupuncture, and sham moxibustion.

#### 2.2.3 Type of outcomes

##### 2.2.3.1 Primary outcomes

The primary outcome of the study is the improvement of cognitive function, as measured by validated assessment tools. The assessment tools include the Mini-Mental State of Examination (MMSE),^[32]^ Montreal Cognitive Assessment (MoCA),^[33]^ Global Deterioration Scale (GDS), and Clinical Memory Scale (CMS).^[34]^

##### 2.2.3.2 Secondary outcomes

Secondary outcomes include changes in the ADLs,^[35]^ clinical efficacy, and the incidences of adverse events related to acupuncture, moxibustion, CHM, and TC (such as allergy, scalding, local infection, coughing, and nausea).

#### 2.2.4 Study design

This study is a systematic review and network meta-analysis of RCTs with TCM therapies on MCI. This research will include all relevant RCTs using TCM therapies for MCI and the first period in randomized crossover trials, regardless of publication status. Quasi-RCTs, animal trails, review documents, clinical experience, and case reports will be excluded. Moreover, we will only search English and Chinese literature in this study. And we will remove the studies without comparable baselines and duplicate publications.

### 2.3 Literature retrieval strategy

Computer retrieval of published RCTs of TCM for MCI is conducted in PubMed, the Cochrane Library (issue 8, 2020), EMbase, China National Knowledge Infrastructure (CNKI), China Biological Medicine (CBM), Chongqing VIP, and Wan-fang databases. The time limit of document retrieval is from the establishment of each database to August 31, 2020. Medical subject heading (MeSH) terms and key words are used to identify RCTs with the limitation of Chinese and English language. In addition, inclusive literature from the field and references from previous evaluations will be manually retrieved to find other potentially relevant articles. Chinese search terms mainly include “mild cognitive impairment”; English search words include “Mild cognitive impairment,” “MCI,” “acupuncture,” “moxibustion,” “Chinese herbal medicines,” “Tai Chi,” etc. Taking PubMed as an example, the initial retrieval strategy is summarized in Table 1 and will be adjusted according to the specific database.

**Table 1:**
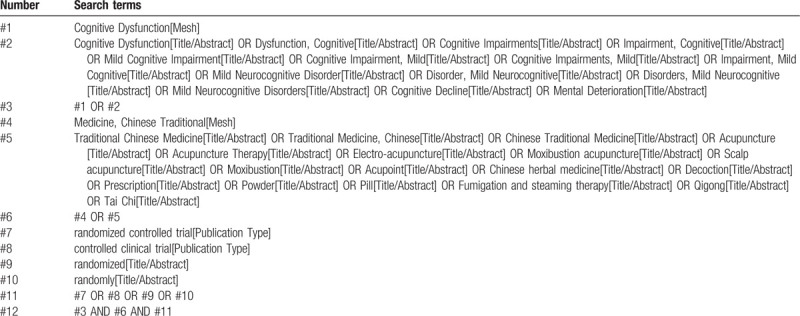
PubMed search strategy.

### 2.4 Literature selection and data extraction

**Figure 1:**
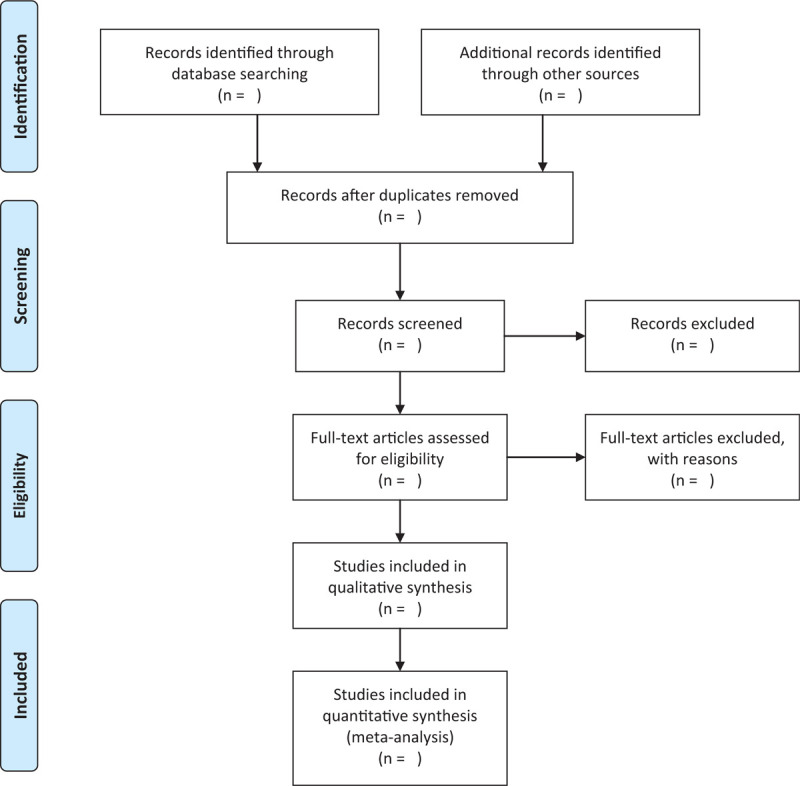
Flow chart of literature screening.

The study selection program will follow the Prisma guidelines, As shown in Fig. 1, Haiyan Wang and Haiyang Yu will independently screen literatures according to inclusion and exclusion criteria and cross-checked against preliminary screening of the literature through Endnote software to remove duplicates; by reading the title and preliminary screening the abstract, exclude the literature that obviously does not meet the inclusion criteria; download and read the full text for re-screening. At the end of the filtering, the extracted features are recorded using a pre-designed data table. These features include title, journal, author, publication year, country, sample size, gender, mean age, intervention, comparator, course of treatment, outcome measures, and follow-up time. If there is any disagreement, the third researcher Kai Song will be asked to assist in the judgment. At the same time, the key factors of bias risk assessment are extracted.

### 2.5 Quality assessment

The quality of systematic review reflects the risk of bias or validity in its process and results, as well as the reliability of the included studies. The quality of the included studies will be assessed according to the Cochrane Reviewers Handbook. Two trained researchers Haiyan Wang and Kai Song will independently evaluate the risk of bias of the included studies. If the results are disputed, they will be submitted to the corresponding author (Hong Zhang) of this study for review and determination.

Cochrane Reviewers’ Handbook will be used to assess the risk of RCTs being included in NMA, including^[36]^ random sequence generation; allocation concealment; blinding of the subjects and researchers; blinding of outcome assessment; incomplete outcome data; selective reporting; other bias.

### 2.6 Data synthesis and statistical methods

#### 2.6.1 Network meta-analysis

This study uses ADDIS 1.16.8 based on Bayesian framework for NMA.^[37]^ Odds ratios (ORs) or standardized mean differences (SMD) will be modeled using Markov chain Monte Carlo methods, both with 95% confidence intervals (CIs). Preset model parameters: 4 chains are used for simulation analysis, with an initial value of 2.5, a step size of 10, 20,000 annealing times, and 50,000 simulation iterations. The network evidence plot will be generated according to different outcome. According to the results of the NMA, rank probability plot of various TCM therapies will be generated and sorted by dominance, with Rank1 being the optimal sort.

#### 2.6.2 Consistency assessments/statistical model selection

The Node-split model is used to check for consistency between direct and indirect evidence. If there is no statistical difference (*P* > .05) between direct comparison and indirect comparison, the consistency model is used, whereas the inconsistency model is used for analysis. If the consistency model is adopted, then the stability of the results is verified by the inconsistency model: when the inconsistency factors including 0, at the same time, inconsistency standard deviation including 1 says the result of consistency model is more stable and reliable. At the same time, various analysis models are iterated with preset parameters, and the convergence of iteration effect is judged by potential scale reduced factor (PSRF). When the PSRF value is close to or equal to 1 (1≤PSRF≤1.05), the convergence is complete, the model has good stability, and the conclusion of analysis is reliable. If the PSRF value is not in this range, the iteration continues manually until the PSRF value reaches the range standard.

#### 2.6.3 Heterogeneity test

Before the combination of effect size, we will use Stata to assess available study and patient characteristics to ensure similarity and to investigate the potential effect of heterogeneity on effect estimates. When interstudy heterogeneity exists, the random effect model is used. For comparison of each pair, heterogeneity is assessed by the statistic *I*^2^ value. When *I*^2^ > 50%, it indicates that there is heterogeneity between studies, and the source of heterogeneity should be further searched. When *I*^2^ < 50%, interstudy heterogeneity is considered to be small or there is no obvious heterogeneity.

#### 2.6.4 Sensitivity analysis

If necessary, the sensitivity analysis will be used to assess the effect of each study on the random effects model. The sensitivity of the general combined effect of all outcome indicators is analyzed by the exclusion method. That is, each study is excluded, and the remaining studies will be reanalyzed to identify the stability of the results. If there is no qualitative change in the combined effect showed in the results, the results are stable.

#### 2.6.5 Subgroup analysis

If necessary, we will conduct a subgroup analysis of duration of treatment, age, the course of MCI, and research quality.

#### 2.6.6 Small sample effect/publication bias

If 10 or more studies are included in the NMA, a comparison-adjusted funnel plot is developed using Stata to evaluate the presence of small sample effects or publication bias in the intervention network. Descriptive analysis will be carried out through the symmetry of funnel plot. If the plot is asymmetric and there is no inverted funnel shape, it indicates that there may be publication bias. This may be related to the difficulty in the publication of the literature with negative results and the low quality of the inclusion methods.

#### 2.6.7 Dealing with missing data

If the required data are lost or incomplete, we will contact the corresponding author of the original document or the relevant email address of the first author. If there is no response, the record is excluded.

#### 2.6.8 Evaluating the quality of the evidence

To grade evidence quality and understand the current situation of evidence rating thereby analyzing possible problems, The Grading of Recommendations Assessment, Development and Evaluation (GRADE) instrument will be used to assess the quality of evidence in the NMA.^[38]^ On the basis of the risk of bias, inconsistency, imprecision, indirection, and publication bias, GRADE grades evidence quality into 4 levels: high, medium, low, and very low.

## 3 Discussion

Dementia is characterized by progressive cognitive decline and is an increasingly common phenomenon within our aging population.^[39]^ Around the world, nearly 25 million people have been diagnosed with dementia, which is a major public health problem.^[40]^ In recent years, with the increasing trend of an aging population, the prevalence of dementia has increased year by year. To prevent or slow the progression of dementia, it is important to manage it in its early stage. However, there are no recommended medications for MCI because cholinesterase inhibitors, well-known antidementia drugs, have more adverse effects than benefits when prescribed to patients with MCI.^[10,11]^ In this situation, many people tend to use complementary and alternative therapies including TCM to treat MCI. TCM therapy could have an indicative role in reducing the cases of MCI or dementia. Therefore, we have to evaluate the efficacy and safety of TCM treatment.^[12]^

We will assess the quality of evidence with the GRADE framework: risk of bias, heterogeneity or inconsistency, imprecision, indirectness, and publication bias. Two independent review authors (HYW and KS) will judge the quality of evidence contributing to primary outcomes (high, moderate, low, or very low), while disagreements will be resolved by discussion with a third member (HZ). Our study will generate evidence of TCM in the treatment of MCI and help to reduce the uncertainty about the effectiveness of MCI management. The results will encourage further suggestions for TCM clinical practice or guideline.

This study has a number of limitations. First, few RCTs comparing interventions and controls were available, limiting the number of studies that could be included in the meta-analysis. Second, a few included reports were therapies, which cannot be blinded to participants, especially acupuncture. However, blinding of outcome assessment and single-blind methodologies should be used where possible to reduce the potential for any biases. In addition, the review may be susceptible to publication bias, though this was not evident when funnel plots were examined. As reported, data were markedly heterogeneous with a significant amount of unreported data.

## Author contributions

**Conceptualization:** Haiyan Wang, Haiyang Yu, Kai Song.

**Data curation:** Haiyan Wang, Kai Song, Fanjie Xiong M. Med.

**Formal analysis:** Fanjie Xiong M. Med.

**Funding acquisition:** Hong Zhang.

**Methodology:** Haiyan Wang, Haiyang Yu, Kai Song.

**Project administration:** Haiyang Yu, Fanjie Xiong M. Med.

**Writing – original draft**: Haiyan Wang, Kai Song.

**Writing – review & editing:** Hong Zhang.

## References

[1] Petersen RC Caracciolo B Brayne C Mild cognitive impairment: a concept in evolution. J Intern Med 2014;275:214 28. 24605806 10.1111/joim.12190 PMC3967548

[2] Levey A Lah J Goldstein F Mild cognitive impairment: an opportunity to identify patients at high risk for progression to Alzheimer's disease. ClinTher 2006;28:991 1001. 10.1016/j.clinthera.2006.07.006 16990077

[3] Ward A Arrighi HM Michels S Mild cognitive impairment: disparity of incidence and prevalence estimates. Alzheimers Dement 2012;8:14 21. 22265588 10.1016/j.jalz.2011.01.002

[4] Ward A Tardiff S Dye C Rate of conversion from prodromal Alzheimer's disease to Alzheimer's dementia: a systematic review of the literature. Dement Geriatr Cogn Dis Extra 2013;3:320 32. 24174927 10.1159/000354370 PMC3808216

[5] Kwon CY Lee B Suh HW Efficacy and safety of auricular acupuncture for cognitive impairment and dementia: a systematic review. Evid Based Complement Alternat Med 2018;2018:3426078. 29955234 10.1155/2018/3426078 PMC6000857

[6] Manly JJ Tang MX Schupf N Frequency and course of mild cognitive impairment in a multiethnic community. Ann Neurol 2008;63:494 506. 18300306 10.1002/ana.21326 PMC2375143

[7] Panza F D’introno A Colacicco AM Current epidemiology of mild cognitive impairment and other predementia syndromes. Am J Geriatr Psychiatry 2005;13:633 44. 16085779 10.1176/appi.ajgp.13.8.633

[8] Roberts RO Knopman DS Mielke MM Higher risk of progression to dementia in mild cognitive impairment cases who revert to normal. Neurology 2014;82:317 25. 24353333 10.1212/WNL.0000000000000055 PMC3929198

[9] Mitchell AJ Beaumont H Ferguson D Risk of dementia and mild cognitive impairment in older people with subjective memory complaints: meta-analysis. Acta Psychiatr Scand 2014;130:439 51. 25219393 10.1111/acps.12336

[10] Vega JN Newhouse PA Mild cognitive impairment: diagnosis, longitudinal course, and emerging treatments. Curr Psychiatry Rep 2014;16:490. 25160795 10.1007/s11920-014-0490-8 PMC4169219

[11] Russ TC Morling JR Cholinesterase inhibitors for mild cognitive impairment. Cochrane Database Syst Rev 2012;9:CD009132. 10.1002/14651858.CD009132.pub2 PMC6464825 22972133

[12] Tricco AC Soobiah C Berliner S Efficacy and safety of cognitive enhancers for patients with mild cognitive impairment: a systematic review and meta-analysis. CMAJ 2013;185:1393 401. 24043661 10.1503/cmaj.130451 PMC3826344

[13] Deng M Wang XF Acupuncture for amnestic mild cognitive impairment: a meta-analysis of randomized controlled trials. Acupunct Med 2016;34:342 8. 27491382 10.1136/acupmed-2015-010989

[14] Cao H Wang Y Chang D Acupuncture for vascular mild cognitive impairment: a systematic review of randomized controlled trials. Acupunct Med 2013;31:368 74. 24123487 10.1136/acupmed-2013-010363 PMC3888636

[15] Zhou J Peng WN Xu M The effectiveness and safety of acupuncture for patients with Alzheimer disease: a systematic review and meta-analysis of randomized controlled trials. Medicine (Baltimore) 2015;94:e933. 26039131 10.1097/MD.0000000000000933 PMC4616366

[16] Zhang X Wu B Nie K Effects of acupuncture on declined cerebral blood flow, impaired mitochondrial respiratory function and oxidative stress in multi-infarct dementia rats. Neurochem Int 2014;65:23 9. 24361538 10.1016/j.neuint.2013.12.004

[17] Lai X Ren J Lu Y Effects of acupuncture at HT7 on glucose metabolism in a rat model of Alzheimer's disease: an 18F-FDG-PET study. Acupunct Med 2016;34:215 22. 26654890 10.1136/acupmed-2015-010865 PMC4941154

[18] Shen X Ding G Wei J An infrared radiation study of the biophysical characteristics of traditional moxibustion. Complement Ther Med 2006;14:213 9. 16911902 10.1016/j.ctim.2005.09.003

[19] Okada K Kawakita K Analgesic action of acupuncture and moxibustion: a review of unique approaches in Japan. Evid Based Complement Alternat Med 2009;6:11 7. 10.1093/ecam/nem090 PMC2644273 18955231

[20] Kawakita K Shinbara H Imai K How do acupuncture and moxibustion act? -Focusing on the progress in Japanese acupuncture research. J Pharmacol Sci 2006;100:443 59. 16799260 10.1254/jphs.crj06004x

[21] Huang Z Qin Z Yao Q Moxibustion for chemotherapy-induced nausea and vomiting: a systematic review and meta-analysis. Evid Based Complement Alternat Med 2017;2017:1 1. 10.1155/2017/9854893 PMC5660813 29234451

[22] Zhang T Wang LP Wang GL Effects of moxibustion on symptoms of mild cognitive impairment: protocol of a systematic review and meta-analysis. BMJ Open 2020;10:e033910. 10.1136/bmjopen-2019-033910 PMC7213842 32350012

[23] Choe S Cai M Jerng UM The efficacy and underlying mechanism of moxibustion in preventing cognitive impairment: a systematic review of animal studies. Exp Neurobiol 2018;27:1 5. 29535565 10.5607/en.2018.27.1.1 PMC5840457

[24] Choi JG Moon M Jeong HU Cistanches Herba enhances learning and memory by inducing nerve growth factor. Behav Brain Res 2011;216:652 8. 20849880 10.1016/j.bbr.2010.09.008

[25] Ahn SM Kim YR Kim HN Beneficial effects of Polygonum multiflorum on hippocampal neuronal cells and mouse focal cerebral ischemia. Am J Chin Med 2015;43:637 51. 26119951 10.1142/S0192415X15500391

[26] Zhang J Liu Z Zhang H A two-year treatment of amnestic mild cognitive impairment using a compound Chinese medicine: a placebo controlled randomized trial. Sci Rep 2016;6:28982. 27373556 10.1038/srep28982 PMC4931444

[27] Klein PJ Adams WD Comprehensive therapeutic benefifits of Taiji: a critical review. Am J Phys Med Rehabil 2004;83:735 45. 15314540 10.1097/01.phm.0000137317.98890.74

[28] Sungkarat S Boripuntakul S Kumfu S Tai Chi improves cognition and plasma BDNF in older adults with mild cognitive impairment: a randomized controlled trial. Neurorehabil Neural Repair 2018;32:142 9. 29353543 10.1177/1545968317753682

[29] Zheng W Xiang YQ Ungvari GS Tai Chi for mild cognitive impairment: a systematic review. Psychogeriatrics 2017;17:514 6. 28703397 10.1111/psyg.12269

[30] Miller SM Taylor-Piliae RE Effects of Tai Chi on cognitive function in community-dwelling older adults: a review. Geriatr Nurs 2014;35:9 19. 24252560 10.1016/j.gerinurse.2013.10.013

[31] Shamseer L Moher D Clarke M Preferred reporting items for systematic review and meta-analysis protocols (PRISMA-P) 2015: elaboration and explanation [published correction appears in. BMJ 2015;350:g7647. 25555855 10.1136/bmj.g7647

[32] Arevalo-Rodriguez I Smailagic N Roqué I Mini-Mental State Examination (MMSE) for the detection of Alzheimer's disease and other dementias in people withmild cognitive impairment (MCI). Cochrane Database Syst Rev 2015;2015:CD010783. 10.1002/14651858.CD010783.pub2 PMC6464748 25740785

[33] Lu J Li D Li F Montreal cognitive assessment in detecting cognitive impairment in Chinese elderly individuals: a population-based study. J Geriatr Psychiatry Neurol 2011;24:184 90. 22228824 10.1177/0891988711422528

[34] Stella F Laks J Govone JS Association of neuropsychiatric syndromes with global clinical deterioration in Alzheimer's disease patients. Int Psychogeriatr 2016;28:779 86. 26673910 10.1017/S1041610215002069

[35] Bruderer-Hofstetter M Sikkes SAM Münzer T Niedermann K Development of a model on factors affecting instrumental activities of daily living in people with mild cognitive impairment: a Delphi study. BMC Neurol 2020;20:10.1186/s12883-020-01843-9 PMC7329426 32611388

[36] Higgins JPT Green S Cochrane Handbook for Systematic Reviews of Interventions Version 5.1.0 [updated March 2011]. The Cochrane Collaboration; 2011.

[37] Van Valkenhoef G Tervonen T Zwinkels T ADDIS: a decision support system for evidence-based medicine. Decision Support Syst 2013;55:459 75.

[38] Guyatt G Oxman AD Akl EA GRADE guidelines: 1. Introduction-GRADE evidence profiles and summary of findings tables. J Clin Epidemiol 2011;64:383 94. 21195583 10.1016/j.jclinepi.2010.04.026

[39] Shim S-M Song J Kim J-H Conversion pattern and predictive factor of mild cognitive impairment in elderly Koreans. Arch Gerontol Geriatr 2016;64:146 50. 26896864 10.1016/j.archger.2016.02.007

[40] Gentile S Second-generation antipsychotics in dementia: beyond safety concerns. A clinical, systematic review of efficacy data from randomised controlled trials. Psychopharmacology 2010;212:119 29. 20661553 10.1007/s00213-010-1939-z

